# Role of socioeconomic and parental involvement factors on children foundational learning skills based on MICS (2017–2018) data Punjab, Pakistan

**DOI:** 10.1038/s41598-022-13540-3

**Published:** 2022-06-20

**Authors:** Asifa Kamal, Naila Amjad, Uzma Yaqoob, Naz Saud, Muhammad Ijaz, Ilyas Khan, Mulugeta Andualem

**Affiliations:** 1 Department of Statistics, Lahore College for Women University, Lahore, Pakistan; 2 Department of Mathematics and Statistics, The University of Haripur, Haripur, Pakistan; 3 Department of Mathematics, College of Science Al-Zulfi, Majmaah University, Al-Majmaah, 11952 Saudi Arabia; 4 Department of Mathematics, Bonga University, Bonga, Ethiopia

**Keywords:** Viral infection, Viral infection, Humoral immunity, Risk factors, Occupational health

## Abstract

Developing countries lack studies investigated the socioeconomic and parental role on students’ learning skills. This study is helpful to detect bottlenecks in the foundational learning skills (reading skills and numeracy skills) in the education system of Pakistan. Reading skills of children are found better who had no functional disabilities. Mothers with higher education had a significant positive contribution toward children learning skills. Children deprived of books for reading in appropriate language had a negative impact on their reading skills. Rich children had predominantly higher possibilities of good learning skills than poor children. Parents who had not attended children’s school to discuss child progress had a significantly negative effect on children’s numeracy skills. Overall parental involvement in some forms had insignificantly improved children reading and numeracy skills in Punjab, Pakistan.

## Introduction

Access to education is widespread in today’s world but the momentum of progress in its quality is not the same in different countries. Only 250 million children in the world have developed both elementary learning skills i.e. numeracy and reading skills^1^, and 61 countries measured foundational reading skills from children below primary level, between 2005 and 2013^2^. Reading and understanding a simple text are basic reading skills. Several local evaluation tests like the Latin American Laboratory for Assessment of the Quality of Education (LLECE), the Programme for the Analysis of Education Systems (PASEC), and the Southern and Eastern Africa Consortium for Monitoring Educational Quality (SACMEQ) had shown poor response of students regarding basic reading skills for those who had been enrolled for at least 6 years in the school^2–5^. Students were not able to read and understand simple text in many countries^2^. This deficiency becomes a major hindrance in the learning process, in the future years of children’s schooling^2^. This deficiency can increase the dropout rate of children from school.

Childhood learning skills are important in the context of human development. Early childhood is a significant period in life in which children learn through socialization with peers, parents, and teachers, and the effects of this learning remain long-lasting in their lives^6^. Reading skills comprised of the development of foundational reading skills and reading comprehension skills.

Mathematics is called the mother of all sciences. Due to the emergence of computers in modern life, good mathematical skills are required for many jobs. Children should have a solid base regarding numeracy skills that would help them to excel in their academic life^2^.

According to the theory of cultural capital, social and traditional norms of family and household atmosphere governed the ambitions and attainments of children related to education^7^. Families enriched with cultural assets are more conscious of school rules and regulations. They generate a sense of obedience and nurture children’s goals towards educational attainments and academic excellence. They also assist them in their syllabus^7^.

Lara and Saracostti^8^ studied cluster analyses of 498 parents or guardians that the children showed high academic achievements whose parents have much involvement in their studies while the children whose parents have less involvement showed the less academic achievements.

Zambrana et al.^9^ suggested that Latino’s parents perceptions had much influence on the children home literacy but also their Oral Reading Skills, considering contextual salient factors (i.e., educational attainment, income status, and parent reading proficiency) as well.

Both parental involvement and parental engagement play a vital role in learning skills. Engagement of parents in learning means that parents share the responsibility to help children in achieving learning goals, and provisions of the learning environment at home, school, and community level. Involvement of parents in learning means attending school meetings or events and participating in activities organized by the school. Parental involvement not only improves the grades of children but also creates confidence, socialization skills, and good classroom behaviors^10–15^.

The development of reading skills in children is not the sole responsibility of teachers, generally, both parents and teachers share that responsibility. Normally parents provide the foundation for reading to children in the preschool period. Parents can play a fundamental part in creating interest in reading. The parent’s role as a reading trainer of children starts from the birthday of a child when a child hears the voice of his parents. Parents’ knowledge about the choice of suitable books for children of different ages is required for a true impact on reading skills.

Socioeconomic status of family cover aspects related to the quality of life that encompass prospects and advantages bestowed by the society. Indicators considered to judge the socioeconomic status are generally parental education, income, and employment. It has been proven by research that children from low socioeconomic backgrounds developed learning skills slowly as compared to children of high socioeconomic backgrounds^16^. It is found that underprivileged children have poor memory and physical health. They developed slow cognitive and reading skills with sluggish socioemotional progress. These children lack basic reading skills like phonological recognition, lexicon, and verbal linguistics^17^. School dropout is observed maximum for children who belonged to low socioeconomic status^18^. The academic growth of economically disadvantaged children also turned negative due to the under-resourced school systems in which they study^19^. These schools lack human resources in the shape of well-trained and qualified teachers and necessary physical resources (libraries, computer labs, etc.) used to enhance the learning capacity of children^20^. The impact of the school environment has proven stronger on academic learning as compared to the home environment^19^.

Ni et al.^21^ by using mixed-method analyses, explored that the role of parent’s involvement is very important in enhancing the family reading environment. Slicker et al.^22^ assessed that parent- child home activity played an important role on children’s early reading and mathematical skills. They used the latent profile analysis (LPA) for early childhood longitudinal study for a kindergarten class and examined those children with the most home activities profile along with very high expectations have the most advanced academic skills.

No studies can be found that assessed the parental role on students’ academic performance in developing countries due to the unavailability of relevant data. There is a need to fill the gap and explore the level of children’s learning skills in Pakistan. Realizing the importance of data on academic skills, the Multiple Indicator Cluster Survey (MICS) has introduced a module to collect data on the learning skills of children. It is necessary to formulate effective data-driven policies to improvement in children’s basic learning skills. Due to the comparative nature of this data, it is helpful to monitor the progress of different countries, particularly UNICEF program countries in attaining the sustainable development goals (SDG 4.1) related to education, considering the different spectrum of learning like parental involvement in children learning process. Data collected by MICS on learning skills is also important to detect bottlenecks in the foundational learning skills of the children^2^.

### Objective of study

The objective of this research is to investigate the socio-economic, demographic, and parental involvement factors that contribute to child learning in Punjab, Pakistan.

## Data and methodology

In the current study, secondary data was used that has been taken from the Multiple Indicator Cluster Survey (MICS) 2017–2018, collected by the Punjab Bureau of Statistics in collaboration with UNICEF^23^. Data were collected from 36 districts of Punjab. Stratification was done on the basis of urban/rural areas. The household was chosen using two-stage random sampling design^24^. Enumeration Areas (EAs) were selected as the first stage unit (or primary sampling unit) and from these “Enumeration Areas”, a sample of the household was taken as a second stage unit. Census enumeration areas were selected with unequal probability (probability proportional to size) from each stratum. A household list of Census 2017 was used that was obtained from the Pakistan Bureau of Statistics. The total sample comprised 53,480 households. Sample weights were reported in the relevant data files^25^.

Questions relevant to foundational learning skills for the children aged 5–17 years were managed either by the mother or custodian/caretaker. A child of this age was selected randomly from the household^24^.

The analysis is carried out in STATA 15.0 under complex survey commands. Variables used for survey setting are “psu”, “stratum”, “fshweight”. Only children aged 7–14 who were interviewed (yes) for this learning module are included in the current study. After applying the age (7–14) restriction sample was reduced to 17,471 children for learning skill analysis out of 27,870 children. Data used was collected through questionnaires (Household, individual women, and child questionnaires from 5 to 17). All questionnaires are available on the website of the Bureau of Statistics Punjab, Government of Punjab^26^.

### Factors and covariates

Foundational reading skills and numeracy skills are taken as a measure of students learning abilities. Reading skill is aggregated by the results of the literal questions and inferential questions and the numeracy skills is aggregated by the results of the number reading, number discrimination, addition, and pattern recognition.

The two dependent variables foundational reading skills and foundational numeracy skills are made dichotomous or binary variables with categories “yes or no”. Response variable foundational reading skill is computed using three criteria i.e. children’s ability to read 90% of words in a story correctly, correctly answering the three literal comprehension questions, and correctly answering two inferential comprehension questions. If a child had performed all three activities correctly then he was categorized as “yes = having good reading skills “and “no = if he failed to accomplish the target of any of three activities”^26^.

Another response variable is foundational numeracy skills. The student was categorized as having a numeracy skill i.e. “yes” if he/she had successfully completed the four tasks i.e. number reading task, number discrimination task, addition task, and pattern recognition & completion task. Otherwise, he/she is placed into the other category i.e. “no”^26^.

Potential factors affecting foundational learning skills chosen from MICS 2017–2018 data set is the age of the child at beginning of school, sex of child (male, female), child functional disability (yes, no), mother’s education (no, primary, middle, secondary, higher), availability of books for reading in the appropriate language (yes, no), care taker’s functional disability (yes, no), area of residence (rural, urban), division (Lahore, Bahawalpur, DG Khan, Faisalabad, Gujranwala, Multan, Rawalpindi, Sahiwal and Sargodha), wealth index quintile (poor, middle, rich) and six parental involvement factors (PR6: Anyone helps a child in homework; PR9A: Meetings for educational issues; PR9B: Meetings for financial issues; PR10: Received report card; PR11A: Attended school celebrations; PR11B: Attended school to discuss child progress ). Two parental involvement factors i.e. PR7 (School has a governing body in which parents can participate) and PR8 (Attended PTA/SMC meeting in the last 12 months) are not included in the study due to the high percentage of missing values. The wealth index quantile variable is re-categorized into three categories from the five categories as in the numeracy skills model, last category of wealth index (fifth) had not been left with substantial number of values needed to fit the model.

### Statistical analysis

The chi-square test is used to test the association between two response variables i.e. reading skills and numeracy skills with different socioeconomic, demographic, and parental involvement factors. The chi-square statistic is computed as:

$$ \chi^{2} = \mathop \sum \limits_{i} \mathop \sum \limits_{j} \frac{{\left( {n_{ij} - \mu_{ij} } \right)^{2} }}{{\mu_{ij} }} $$

The null hypothesis is rejected in favor of the alternative hypothesis for large values of $\chi^{2}$.

Logistic regression is a mathematical model approach that can be used to describe the relationship of several independent variables to a dependent variable that is binary or dichotomous (yes, no). Model is designed to predict the probability of an event occurring (i.e. the probability of an observation being in the group coded 1). The model has the following form:

$$ {\varvec{Y}}_{{\varvec{i}}} = {{\varvec{\upalpha}}} \, + \, {{\varvec{\upbeta}}}{\varvec{x}}_{{\varvec{i}}} + \varepsilon_{{\varvec{i}}} $$

$$ P(Y|X) = \frac{{e^{{\beta_{o} + \beta_{1} X}} }}{{1 + e^{{\beta_{o} + \beta_{1} X}} }} $$

$$ \ln \left( {\frac{P(Y|X)}{{1 - P(Y|X)}}} \right) = \beta_{o} + \beta_{1} X $$

Generally, the shape of the response function in case of a binary response variable is S-shaped and has the form as given:

$$ E(Y) = \frac{{{\varvec{e}}^{{{\varvec{\alpha}} + \user2{\beta X}}} }}{{1 + \user2{ e}^{{{\varvec{\alpha}} + \user2{\beta X}}} }} $$

Consider the p independent variables denoted by the vector x_i_ = ($x_{1}$_,_$x_{2}$,$x_{3}$,…,$x_{k}$). Let the conditional probability that the outcome is present be denoted by $p$ then the logit of the multiple logistic regression model is given by the equation:

$$ \begin{gathered} \ln \left( {\frac{p}{1 - p}} \right) = \beta_{o} + \beta_{1} X_{1} + \beta_{2} X_{2} + \cdots + \beta_{p} X_{p} \hfill \\ {\mathbf{g}}\left( {\mathbf{x}} \right) \, = {\varvec{\beta}}_{{\varvec{o}}} + {\varvec{\beta}}_{1} {\varvec{x}}_{1} + \cdots + {\varvec{\beta}}_{{\varvec{p}}} {\varvec{x}}_{{\varvec{p}}} \hfill \\ \end{gathered} $$

## Results

**Figure 1:**
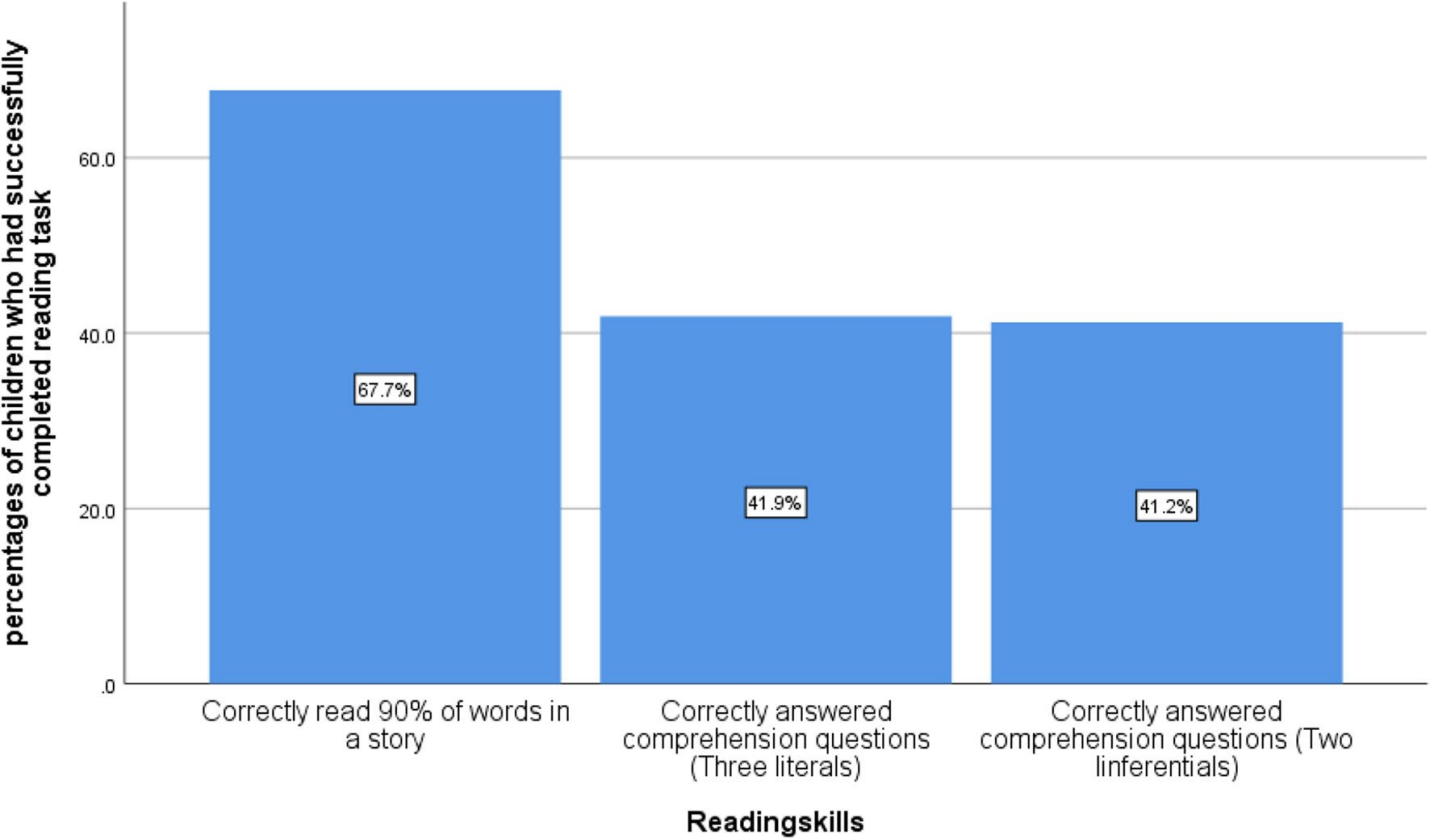
Foundational Reading Skills of Children of Age 5–17 Years of Punjab. Source: MICS 2017–18.

From Fig. 1 it has been observed that 67.7% children of ages 7–14 years can read 90% of words in a story. The percentage of children who had correctly answered three literal comprehension questions is 41.9%. The percentage of children who had correctly answered two inferential comprehension questions is found 41.2%.

**Figure 2:**
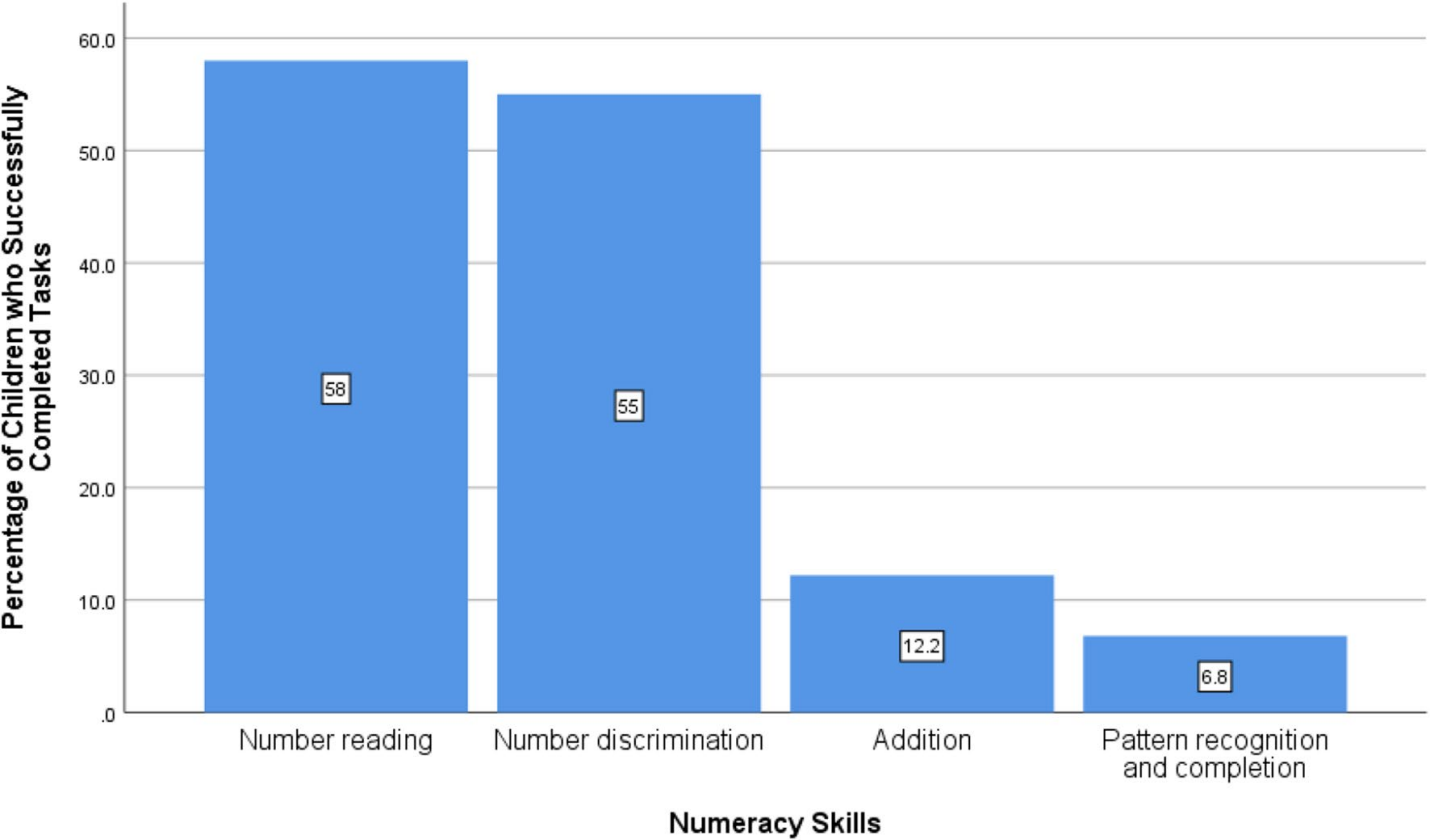
Foundational Numeracy skills of Children of Age 5–17 Years of Punjab. Source: MICS 2017–18.

It can be seen from Fig. 2 that the percentage of children of ages 7–14 years who had accurately read numbers is 58%. It has also been observed from the graph that 55% of children had number discrimination skills and 12.2% of children had addition skills. Only 6.8% of children were successful in pattern recognition and completion skill.

**Figure 3:**
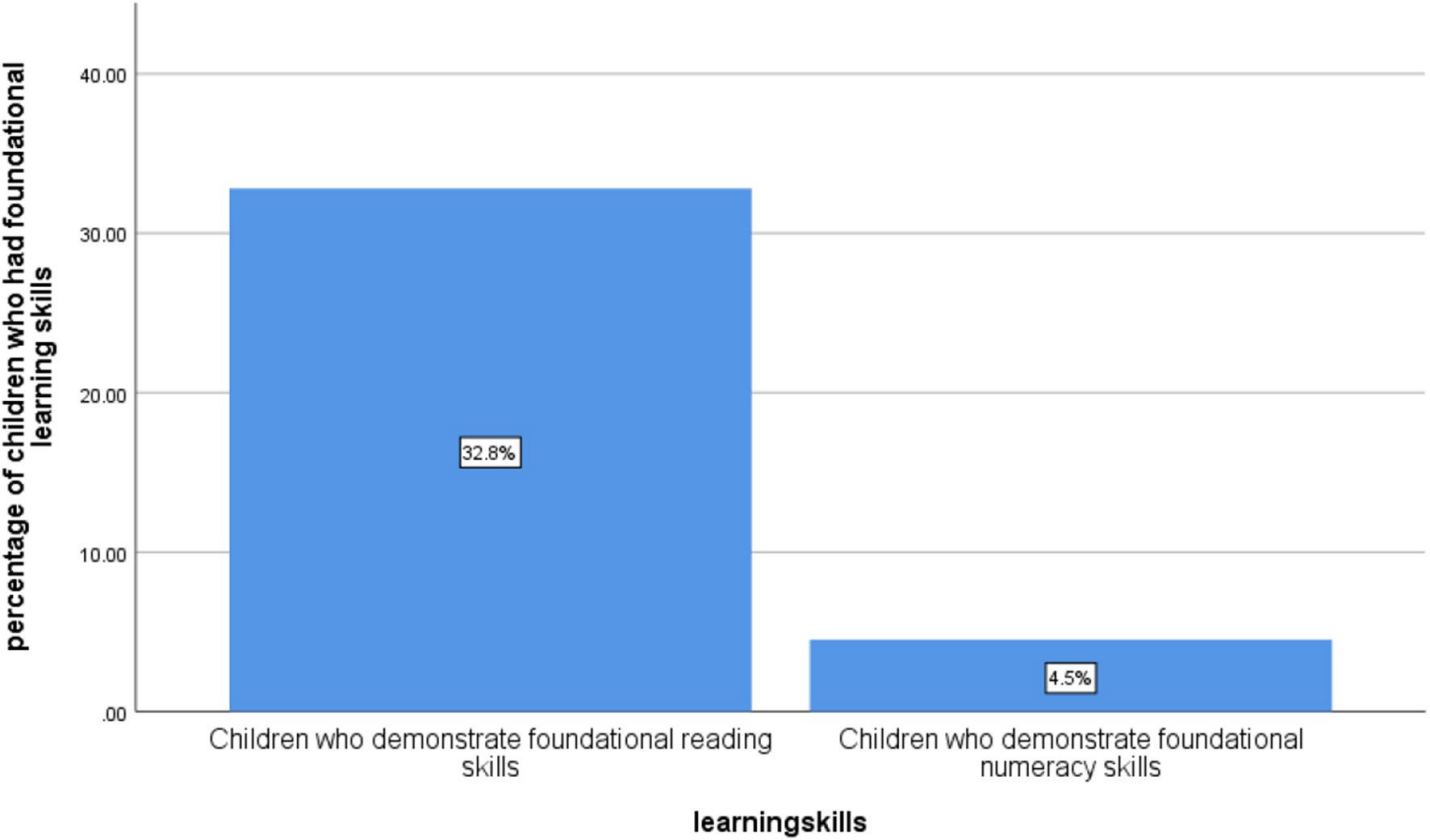
Foundational Learning skills of Children of Age 5–17 Years of Punjab. Source: MICS 2017–18.

Overall, only 4.5 and 32.8% of children had demonstrated foundational numeracy skills and foundational reading skills respectively in Punjab (Fig. 3).

All background characteristics of children except gender have shown significant association with numeracy skills and reading skills (Table 1). It has been observed from Table 1 that there is little difference in the percentage of male and female children regarding adequate numeracy skills and reading skills. An increasing pattern in the percentage of children is evident for both adequate numeracy skills (yes) and adequate reading skills (yes) with an increase in the age of children at the beginning of school till age 11 years. After age 11 years, there is a negligible decline in the percentage for numeracy skills, accompanied by a sudden increase in the percentage for children aged 14 years. This shows that learning skills improved with age increase in age at the beginning of the school of the child. But after 11 or 12 years, the increase turned into a decrease. Surprisingly, the percentage of children having sufficient numeracy skills is higher for those children who had any functional disability (4.72%) as compared to those children that had not any functional disability (3.53%). The percentage of children with satisfactory reading skills is higher for those who had no functional disability (37%) as compared to those who had a functional disability (30.1%). A similarly rising trend in the percentage of children who had ample numeracy skills and reading skills is found with an increase in the level of maternal education. The improvement in reading skills or numeracy skills went consistently higher with rising in maternal education level. The percentage of children who had adequate reading skills is higher for children for whom reading books is available in the appropriate language (37.4%). The percentage of children who had necessary reading skills is almost the same irrespective of care taker’s disability.

**Table 1:** Percentage Distribution of Children (Learning Skills × Background Characteristics) in MICS 2018, Punjab.

		Foundational numeracy skills			Foundational reading skills		
Background characteristics		Yes	No	$\chi^{2}$	Yes	No	$\chi^{2}$
Sex	Male	4.83%	95.2%	8.6404 (0.0542)	35.2%	64.8%	6.8896 (0.0994)
	Female	4.19%	95.8%		36.5%	63.5%	
Functional disability	Yes	4.72%	95.3%	17.2466 (0.0067)	30.1%	69.9%	107.7606 (0.0000)
	No	3.53%	96.5%		37%	63%	
Age at the beginning of school	6	1.21%	98.8%	354.3700 (0.0000)	10.1%	89.9%	4056.4600 (0.0000)
	7	2.52%	97.5%		17.8%	82.2%	
	8	3.3%	96.7%		24.9%	75.1%	
	9	4.59%	95.4%		38.7%	61.3%	
	10	5.35%	94.7%		46.1%	53.9%	
	11	6.45%	93.6%		47.9%	52.1%	
	12	6.25%	93.8%		53.5%	46.5%	
	13	6.21%	93.8%		52.6%	47.4%	
	14	10%	90%		52.2%	47.8%	
Mother’s education	No/Pre School	3.54%	96.5%	213.4513 (0.0000)	26.4%	73.6%	2153.8531 (0.0000)
	Primary	4.27%	95.7%		38.8%	61.2%	
	Middle	4.81%	95.2%		45.7%	54.3%	
	Secondary	5.83%	94.2%		49.7%	50.3%	
	Higher	8.92%	91.1%		60.5%	39.5%	
Book availability	Yes	–	–	–	37.4%	62.6%	244.987 (0.000)
	No	–	–	–	26.1%	73.9%	
Care taker disability	Yes	2.73%	97.3%	12.5057 (0.0426)	35.6%	64.4%	0.0080 (0.9554)
	No	4.45%	95.5%		35.5%	64.5%	
Wealth index quantile	Poorest	2.9%	97.1%	184.8384 (0.0000)	15.4%	84.6%	3208.4911 (0.0000)
	Second	3.92%	96.1%		30.7%	69.3%	
	Middle	4.44%	95.6%		39.2%	60.8%	
	Fourth	4.59%	95.4%		44.2%	55.8%	
	Richest	7.37%	92.6%		56.8%	43.2%	
Area	Urban	5.95%	94.1%	95.3364 (0.0000)	45.7%	54.3%	839.9999 (0.0000)
	Rural	3.74%	96.3%		30.5%	69.5%	
Division	Lahore	6.21%	93.8%	413.2552 (0.0000)	42.8%	57.2%	473.5467 (0.0000)
	Bahawalpur	2.05%	97.9%		31.2%	68.8%	
	DG Khan	5.63%	94.4%		27.2%	72.8%	
	Faisalabad	3.08%	96.9%		34.5%	65.5%	
	Gujranwala	3.65%	96.4%		39%	61%	
	Multan	4.02%	96%		33.3%	66.7%	
	Rawalpindi	1.7%	98.3%		44.9%	55.1%	
	Sahiwal	5.28%	94.7%		33.1%	66.9%	
	Sargodha	10.2%	89.8%		32.1%	67.9%	

*p*-value < 0.05 (significant), ( ) = *p*-value.

But, the percentage of children who had adequate numeracy skills is higher (4.45%) for those who were cared for by a person without any disability. An increase in the percentage of children who had adequate reading skills or numeracy rises with an increase in the wealth index quintile. The percentage of children with acceptable reading and numeracy skills is higher for urban children as compared to their rural counterparts. The percentage of children with adequate numeracy skills is observed highest for the Sargodha division (10.2%) and lowest for the Rawalpindi division (1.7%). While the percentage of children with fundamental reading skills is highest for the Rawalpindi division (44.9%) and lowest for DG Khan (27.2%).

The percentage of children with adequate learning skills both learning and numeracy skills are observed higher for parents who helped them in doing homework or were involved in academic activities (attended school meetings for educational/financial matters or school celebrations or to receive report card) as compared to children whose parents had not participated in academic activities of their offspring (Table 2).

**Table 2:** Percentage Distribution of Children (Learning Skills × Parental Involvement Factors) in MICS 2018, Punjab.

		Foundational Numeracy skills			Foundational reading skills		
Factors		Yes	No	$\chi^{2}$	Yes	No	$\chi^{2}$
PR6 Any one helps child in home work	Yes No	5.42%	94.6%	5.6197 (0.1777)	45.8%	54.2%	172.5184 (0.0000)
		4.85%	95.2%		38.8%	61.2%	
PR9A Meetings for edu. issues	Yes No	5.22%	94.8%	46.7235 (0.0721)	51.9%	48.1%	95.5941 (0.0061)
		3.19%	96.8%		44.8%	55.2%	
PR9B Meetings for financial issues	Yes No	5.05%	94.9%	10.7114 (0.3828)	55.9%	44.1%	259.2641 (0.0000)
		4.1%	95.9%		44.6%	55.4%	
PR10 Received report card	Yes No	5.64%	94.4%	30.6575 (0.0007)	47.4%	52.6%	722.6414 (0.0000)
		4.3%	95.7%		32.8%	67.2%	
PR11A Attended school celebrations	Yes No	5.59%	94.4%	6.4630 (0.1500)	49.7%	50.3%	369.3389 (0.0000)
		4.92%	95.1%		38.3%	61.7%	
PR11B Attended school to discuss child progress	Yes No	5.46%	94.5%	12.6241 (0.0336)	45.8%	54.2%	380.6454 (0.0000)
		4.6%	95.4%		35.3%	64.7%	

*p*-value < 0.05 (significant), ( ) = *p*-value.

Binary Logistic regression models are fitted independently on the two response variables i.e. adequate reading skills (Table 3) and adequate numeracy skills (Table 4).

**Table 3:** Odd Ratios of Children Background Characteristics and Parental Involvement Factors on Foundational Reading Skills using Logistic Regression, MICS Punjab 2018.

Characteristics	Unadjusted model		Model I		Model II		Model III	
			Children background characteristics		Parental involvement factors		Children background and parental involvement factors	
	OR	95% CI	OR	95% CI	OR	95% CI	OR	95% CI
**Sex (ref. = Male)**	–	–	–	–	–	–	–	–
Female	1.06	0.98–1.13	1.08	0.99–1.17			1.04	0.83–1.32
Age of child at beginning of school year	1.34*	1.32–1.36	1.43*	1.41–1.46	–	–	1.45*	1.37–1.54
**Functional disability (ref. = Yes)**	–	–	–	–	–	–	–	–
No	1.36*	1.24–1.49	1.38*	1.24–1.55			1.95*	1.40–2.71
**Mother’s education (ref. = ** **No/Preschool)**	–	–	–	–	–	–	–	–
Primary	1.77*	1.61–1.93	1.71*	1.53–1.99	–	–	1.28	0.89–1.85
Middle	2.35*	2.08–2.65	1.99*	1.71–2.30	–	–	1.34*	1.07–2.51
Secondary	2.75*	2.47–3.06	2.40*	2.10–2.77	–	–	1.82*	1.23–2.69
Higher	4.27*	3.78–4.82	3.99*	3.42–4.65	–	–	3.13*	2.15–4.56
**Book availability (ref. = Yes)**	–	–	–	–	–	–	–	–
No	0.59*	0.52–0.66	0.60*	0.53–0.68			0.34*	0.24–0.50
**Care taker disability(ref. = Yes)**	–	–	–	–	–	–	–	–
No	1.002	0.85–1.16	0.98	0.82–1.17			1.37	0.77–2.46
**Wealth index quantile (Poor)**	–	–	–	–	–	–	–	–
Middle	2.22*	2.02–2.42	1.87*	1.66–2.10			1.18	0.77–1.79
Rich	3.48*	3.21–3.78	2.34*	2.04–2.67			1.30	0.86–1.97
**Area (ref. = Urban)**	–	–	–	–	–	–	–	–
Rural	0.52*	0.48–0.56	1.00	0.90–1.11			0.87	0.67–1.14
**Division (ref. = Lahore)**	–	–	–	–	–	–	–	–
Bahawalpur	0.61*	0.50–0.72	1.18	0.96–1.45	–	–	4.42*	1.86–10.52
DG Khan	0.50*	0.42–0.58	1.14	0.94–1.37	–	–	1.83	0.84–3.95
Faisalabad	0.70*	0.61–0.81	1.02	0.87–1.20	–	–	1.84*	1.18–2.87
Gujranwala	0.85*	0.75–0.97	0.80*	0.69–0.93	–	–	1.13	0.80–1.60
Multan	0.67*	0.58–0.76	1.21*	1.02–1.43	–	–	1.83*	1.09–3.06
Rawalpindi	1.09	0.93–1.27	1.08	0.89–1.30	–	–	1.78*	1.23–2.60
Sahiwal	0.66*	0.55–0.78	1.21	0.98–1.45	–	–	2.16*	1.12–4.17
Sargodha	0.63*	0.54–0.73	1.08	0.90–1.31	–	–	1.19	0.60–2.37
**PR6 Any one helps child in home work(ref. = Yes)**	–	–	–	–			–	–
No	0.75*	0.69–0.80			0.72*	0.58–0.90	0.79	0.60–1.05
**PR9A Meetings for edu. issues (ref. = Yes)**	–	–	–	–			–	–
No	0.75*	0.61–0.92			0.98	0.77–1.26	1.10	0.83–1.46
**PR9B Meetings for financial issues (ref. = Yes)**	–	–	–	–			–	–
No	0.64*	0.51–0.77			0.72*	0.56–0.92	0.61*	0.46–0.82
**PR10 Received report card (ref. = Yes)**	–	–	–	–			–	–
No	0.54*	0.50–0.58			0.88	0.64–1.22	1.06	0.72–1.55
**PR11A Attended school celebrations (ref. = Yes)**								
No	0.62*	0.58–0.67			0.87	0.70–1.09	0.89	0.68–1.16
**PR11B Attended school to discuss child progress (ref. = Yes)**	–	–	–	–			–	–
No	0.64*	0.59–0.69			0.83	0.65–1.06	0.96	0.70–1.30
**Constant**	–	–	0.01*	0.04–0.01	1.51*	1.25–1.82	0.01*	0.002–0.01

*Significant at 5%, Model I, II and III are adjusted models, edu. = educational.

**Table 4:** Odd Ratios of Children Background Characteristics and Parental Involvement Factors on Foundational Numeracy Skills using Logistic Regression, MICS Punjab 2018.

Characteristics	Unadjusted model		Model I		Model II		Model III	
			Children background characteristics		Parental involvement factors		Children background and parental involvement factors	
	OR	95% CI	OR	95% CI	OR	95% CI	OR	95% CI
**Sex (ref. = Male)**	–	–	–	–	–	–	–	–
Female	0.86	0.74–1.00	0.85	0.72–1.02	–	–	0.92	0.55–1.54
Age of child at beginning of school year	1.21*	1.17–1.25	1.23*	1.19–1.27	–	–	1.16*	1.04–1.30
**Functional disability (ref. = Yes)**	–	–	–	–	–	–	–	–
No	1.35*	1.08–1.68	1.12	0.88–1.42	–	–	1.50	0.61–3.68
**Mother’s education (ref. = No/Preschool)**	–	–	–	–	–	–	–	–
Primary	1.21	0.97–1.50	1.28	1.00–1.67	–	–	1.22	0.39–3.79
Middle	1.38*	1.03–1.83	1.37	0.96–1.95	–	–	0.58	0.14–2.47
Secondary	1.69*	1.31–2.16	1.72*	1.25–2.38	–	–	1.84	0.65–5.21
Higher	2.67*	2.03–3.50	2.54*	1.80–3.60	–	–	4.37*	1.76–10.89
**Care taker disability(ref. = Yes)**	–	–	–	–	–	–	–	–
No	1.66*	1.01–2.73	1.60	0.97–2.66	–	–	1.98	0.24–16.21
**Wealth index quantile (Poor)**	–	–	–	–	–	–	–	–
Middle	1.33*	1.09–1.63	1.31*	1.00–1.72	–	–	5.63	0.60–52.97
Rich	1.81*	1.50–2.18	1.47*	1.05–2.05	–	–	10.51*	1.25–88.59
**Area (ref. = Urban)**	–	–	–	–	–	–	–	–
Rural	0.61*	0.51–0.73	0.90	0.71–1.14	–	–	1.42	0.71–2.85
**Division (ref. = Lahore)**	–	–	–	–	–	–	–	–
Bahawalpur	0.32*	0.20–0.49	0.49*	0.30–0.79	–	–	0.64	0.08–5.32
DG Khan	0.90*	0.64–1.25	1.35	0.94–1.93	–	–	0.78	0.08–7.41
Faisalabad	0.48	0.33–0.69	0.54*	0.37–0.80	–	–	1.37	0.59–3.18
Gujranwala	0.57*	0.39–0.82	0.59*	0.40–0.89	–	–	0.21*	0.06–0.74
Multan	0.63*	0.44–0.89	0.81	0.56–1.17	–	–	0.43	0.11–1.71
Rawalpindi	0.26*	0.17–0.39	0.23*	0.15–0.36	–	–	0.10*	0.03–0.37
Sahiwal	0.84	0.57–1.22	1.23	0.83–1.83	–	–	0.72	0.21–2.42
Sargodha	1.72*	1.27–2.33	2.48*	1.80–3.41	–	–	1.18	0.38–3.66
**PR6 any one helps child in home work(ref. = Yes)**	–	–	–	–	–	–	–	–
No	0.89	0.75–1.05			1.20	0.70–2.05	1.58	0.88–2.86
**PR9A Meetings for edu. issues (ref. = Yes)**	–	–	–	–	–	–	–	–
No	0.60	0.33–1.05			0.63	0.33–1.20	0.72	0.33–1.59
**PR9B Meetings for financial issues (ref. = Yes)**	–	–	–	–	–	–	–	–
No	0.80	0.49–1.31			0.95	0.52–1.72	1.07	0.56–2.03
**PR10 Received report card (ref. = Yes)**	–	–	–	–	–	–	–	–
No	0.75*	0.63–0.88			0.52	0.20–1.38	0.69	0.25–1.91
**PR11A Attended school celebrations (ref. = Yes)**	–	–	–	–	–	–	–	–
No	0.87	0.72–1.05			1.67	0.90–3.10	2.03	1.02–4.04
**PR11B Attended school to discuss child progress (ref. = Yes)**	–	–	–	–	–	–	–	–
No	0.84*	0.71–0.98			0.30*	0.13–0.66	0.40*	0.17–0.93
**Constant**	–	–	0.003*	0.001–0.01	0.05*	0.03–0.09	0.000*	0.000–0.01

*Significant at 5%, Model I, II and III are adjusted models.

Potential socio-economic, demographic factors named child background characteristics (sex of child, age of child at beginning of school, functional disability, mother’s education, availability of book for reading, caretaker disability, area of residence Division and wealth index) and parental involvement factors (anyone helps the child in homework, meetings at school for educational issues, meetings at school for financial issues, received report card of the child, attended school celebrations, attended school to discuss child progress) available in MICS 2017–2018 were used for the current analysis.

The age of the child at the beginning of school remained significant in all models with a slight increase in the value of OR for model III (Table 3). Chances of children having adequate reading skills increase by 43% (OR = 1.43; CI = 1.41, 1.46) and 45% (OR = 1.45; CI = 1.37, 1.54) for per year increase in age of children at beginning of school in the child background characteristic model (Model I) and in the combined model (Model III) respectively.

It is observed that children with no functional disability had significantly higher adequate reading skills (Table 3). In Model I, children with no functional disability had (OR = 1.38; CI = 1.24, 1.55) 1.38 times higher adequate reading skills as compared to children with functional disability. In model III, when controlled also for the parental involvement factor, the odds ratio (OR = 1.95; CI = 1.40, 2.71) is increased.

Mother’s education had also played a significant role in affecting the children learning skills in the current study (Table 3). Odd ratios significantly increase as the maternal level of education increases. Chances of children’s reading skills are observed to be 1.71, 1.99, 2.40, and 3.99 higher for various levels of mother’s education respectively as compared to children of uneducated mothers (Model I). In the combined model, the same trend is observed except that effect turned insignificant in the model for primary educated mothers. Chances of adequate reading skills in children increased thrice (OR = 3.13; CI = 2.15, 4.56) for highly educated mothers as compared to uneducated mothers.

It is evident from Table 3 that the reading skills of children for whom reading books were not available in appropriate language had significantly lower chances (OR = 0.60; CI = 0.53, 0.68) for Model I and Model 111 (OR = 0.34; CI = 0.24, 0.50). Those children who were deprived of reading books in appropriate language had (1–0.34 = 0.66) 66% lower chances of adequate reading ability as compared to their other counterparts.

Wealth index had a significant role in the unadjusted model and child background characteristics model (Model I) but it turned insignificant in the combined model (Model III) when parental involvement factors are entered (Table 3). Chances of children reading abilities were significantly higher for the middle (OR = 1.87; CI = 1.66, 2.10) and rich (OR = 2.34; CI = 2.04, 2.67) background children as compared to poor children. The effect turned insignificant in Model III when parental involvement factors were introduced in the model.

The impact of the area of residence is significant only in the unadjusted model (Table 3). Rural children had (1–0.52 = 0.48) 48% lesser chances of adequate reading skills as compared to urban residents. When controlled for other factors (Model I, Model III) it had lost its significance. Area of residence (rural) is only significant in unadjusted reading skills model (OR = 0.52; CI = 0.48, 0.56) and numeracy skills model (OR = 0.61; CI = 1.41, 1.46).

Lahore has been taken as a reference category for the factor “division”. Children who belonged to the other eight divisions except Rawalpindi had significantly lower reading skills as compared to children from the Lahore division (Table 3). The significance of the model is retained only for Gujranwala (OR = 0.80; CI = 0.69, 0.93) and Multan (OR = 1.21; CI = 1.02, 1.43) division in the child background model (Model I). When parental involvement factors are incorporated in model III, Bahawalpur (OR = 4.42; CI = 1.86, 10.52), Faisalabad (OR = 1.84; CI = 1.18, 2.87), Multan (OR = 1.83; CI = 1.09, 3.06), Rawalpindi (OR = 1.78; CI = 1.23, 2.60) and Sahiwal (OR = 2.16; CI = 1.12, 4.17) division become significant. Children who belonged to the Gujranwala division had (1–0.80 = 0.20) 20% lower chances of adequate reading skills as compared to children living in Lahore when adjusted for child background characteristics. After adjusting for parental involvement factors, children who lived in other divisions had higher chances of adequate reading skills as compared to children who belonged to Lahore.

From parental involvement factors, children whose parents did not help them in doing homework is significant in the unadjusted model (OR = 0.75; CI = 0.69, 0.80) and also in the parental involvement factor model (OR = 0.72; CI = 0.58, 0.90) of reading skills (Table 3). Children whose parents had not helped them in doing homework had 28% lower chances (1–0.72 = 0.28) of adequate reading skills as compared to those children whose parents helped them in doing so. Parents who had not attended meetings at school in the last 12 months to discuss educational issues of child had significantly lower chances (OR = 0.75; CI = 0.61, 0.92) i.e. 25% less probability of adequate reading skills as compared to children whose parents had attended such meetings in an unadjusted model only (Table 3). This factor turned insignificant when controlled for other covariates. Parents who had not attended meetings in the last 12 months at school to discuss financial issues related to school had reduced chances for the parental involvement model (OR = 0.72; CI = 0.56, 0.92), and combined model (OR = 0.61; CI = 0.46, 0.82) of adequate reading skills of children as compared to children whose parents had not been involved in such activities. The odds are 28% (1–0.72 = 0.28) and 39% (1–0.61 = 0.39) less for children whose parents had not attended meetings with school authorities related to financial issues as compared to parents who had attended these types of meetings in the last 12 months for model II and III respectively. This factor is significant only in the unadjusted model of reading skills (Table 3). Children whose parents had not received report cards had significantly lesser chances (OR = 0.54; CI = 0.50, 0.58) of adequate reading skills as compared to children whose parents visited the school to receive report cards.

There are 38% (1–0.62 = 0.38) reduced chances for adequate reading skills for children whose parents had not attended school celebrations of their children as compared to those children whose parents had attended as observed from the unadjusted model of reading skills (Table 3). For other models, this factor turned insignificant when controlled for child background characteristics or parental involvement factors.

It is evident from the unadjusted model that about 36% (OR = 0.64; CI = 0.59, 0.69) reduction in the chances of adequate reading skills in children whose parents had not attended school to discuss the children’s progress has been found as compared to children whose parents’ attended meetings for the aforesaid purpose (Table 3). This factor is no more significant in the rest of the models of reading skills when controls are introduced.

The age of child at the beginning of school has also a thoroughly significant effect in all models (unadjusted, Model I and Model III) computed for numeracy skills. When controlled for background characteristics of a child there are 23% (OR = 1.23; CI = 1.19, 1.27) elevated possibility that the child attained adequate numeracy skills target for per year increase in his age at the beginning of school (Table 4). The odds slightly decreased (OR = 1.16; CI = 1.04, 1.30) in the combined model but the trend and significance remained the same.

It is more likely that children with no functional disability had a higher probability of adequate numeracy skills but the impact is significant (OR = 1.35; CI = 1.08, 1.68) in only the unadjusted model (Table 4). Caretakers of the child without any functional disability had a significantly (OR = 1.66; CI = 1.01, 2.73) positive impact on adequate numeracy skills only in the unadjusted model (Model I).

Mothers with secondary (OR = 1.72; CI = 1.25, 2.38) and higher education (OR = 2.54; CI = 1.80, 3.60) had significantly higher chances that their children possess adequate numeracy skills as compared to uneducated mothers (Model I). In Model III, children of only highly educated mothers had shown significantly higher chances of adequate numeracy skills i.e. 4.37 times more as compared to children of uneducated mothers.

For numeracy skills (Table 4), children of the middle class (OR = 1.31; CI = 1.00, 1.72) and the rich class (OR = 1.47; CI = 1.05, 2.05) had higher chances (OR = 1.31, 1.47) of adequate numeracy skills as compared to poor children (Model I). In the combined model, when adjusted for both child background characteristics and parental involvement factors, only rich children had significantly higher odds (OR = 10.51; CI = 1.25, 88.59) of adequate numeracy skills as compared to poor children. This is the highest odd ratio observed in all models. Chances of adequate numeracy skills are observed lower for rural children as compared to urban (Table 4).

In the numeracy skills model (Table 4), adjusted for child background characteristics, children living in Bahawalpur (OR = 0.49; CI = 0.30, 0.79), Faisalabad (OR = 0.54; CI = 0.37, 0.80), Gujranwala (OR = 0.59; CI = 1.40, 0.89) and Rawalpindi (OR = 0.23; CI = 0.15, 0.36) had significantly fewer chances of adequate numeracy skills as compared to children who lived in Lahore. Only children from Sargodha (OR = 2.48; CI = 1.80, 3.41) division had a probability of higher adequate numeracy skills as compared to children living in Lahore. In the combined model (Model III), children living in Gujranwala had 79% (OR = 0.21; CI = 0.06, 0.74), Rawalpindi 90% (OR = 0.10; CI = 0.03, 0.37) fewer chances of adequate numeracy skills as compared to children living in Lahore.

Children whose parents had not received report cards had 25% (OR = 0.75; CI = 0.63, 0.88) lesser chances of adequate numeracy skills as compared to those children whose parents had received report cards for their children (Table 4).

For numeracy skills models (Table 4), children whose parents had not attended school to discuss the progress of children is thoroughly significant in all unadjusted and adjusted models. In the parental involvement model and combined model, there are 70% (OR = 0.30; CI = 0.13,0.66) and 60% (OR = 0.40; CI = 0.17,0.93) respectively lesser chances that children have adequate numeracy skills if their parents had not attended school to discuss their performance in studies as compared to children whose parents discussed their progress with school teachers.

## Discussion

The average age of a child entering into school varies country-wise. The average age for the beginning of school in European countries varies from 4 to 7 years^27^. Many countries in the world are in line with European policy and appreciate that age at beginning of school should be 6 years^27^. In Pakistan, many parents send their children to school at three years of age. These children are deprived of their parents’ love and attention and thus lack confidence. In the current study, the age of a child at the beginning of school started from six. Age has been observed significantly positively related to children learning skills due to the natural mental development of children. It is also observed in the current study that too much delay (after age 10 years) in starting school has a negative impact on learning skills. It has been observed through research that children enrolled in the younger age group performed less well as compared to their older mates.

It is obvious from the study that the performance of male children is better for foundational numeracy skills as compared to female children in Pakistan. Lindberg, Hyde, Petersen, and Linn^28^ also found that the gender difference in mathematics is not significant. On contrary, female children performed well as compared to male children for basic reading skills. Gender difference in reading skills was observed in every country that participated in PISA (Programme for International Student Assessment) in 2009^29^. In that study, girls outperform boys in reading scores in 14 countries with a difference of more than 50 scores. Popular theories to reason out this gender gap in reading are biological and socio-cultural^30^. Biological theories explain that difference is due to brain wiring, maturity level, and chemistry of boys. Many contradictory reasons are popular in the literature that explains why boys outperform in mathematics as compared to girls^31^. Cognitive and social reasons are two of these. Consequence of this difference in academic performance is that it may determine the choice of subjects in their higher studies. Boys may prefer more STEM subjects while girls do not. Girls tend more toward literature, arts, social science and academic disciplines.

Functional disability had hit the children reading ability more than numeracy skills in the current study. Literacy skills can be affected by learning disabilities e.g. vision, hearing, or speech impediments. Literature informs that disabled children live in different personal situations from their non-disabled peers, and are more likely to experience higher levels of poverty and personal and social disadvantage than other children^32^. Inaccessibility of equipment needed for these children, like hearing and vision aids, electronically adapted mobility devices, and walking frames becomes a continuing barrier to their learning skills. Another vital reason is the unsatisfactory training and professional support required for these children in developing countries like Pakistan.

Parental education has a deep influence on children’s academic performance^33^. Highly educated mothers are surrounded by a social system that is comprised of awareness, talents, and capital that leads to academic success. Maternal education played the dominant role in the current study in accelerating the reading skills of children as compared to uneducated mothers irrespective of their level of education. The impact of higher maternal education is more noticeable in building numeracy skills. The reason might be that mothers with higher education are more likely to render their children to activities that stimulate their numeracy skills. Family income and a mother’s education both have a strong impact on children’s language skills^34^. Englund et al.^35^ found that mothers with higher education can provide more support to their children in problem-solving situations at the preschool level.

Children early reading proficiency is related to the learning atmosphere at home, access to books for reading, and other learning material^19,36^. Radebe^37^ stated that children read for many reasons; to learn, dream, enjoy and explore both the familiar and unknown. Books play an important in developing children reading skills. The availability of books has shown a thoroughly significant effect on children’s basic reading skills in current research.

Limited family resources, enforce poor families to not invest sufficiently in their children’s education that in turn affects their children’s academic accomplishment. Students’ cognitive skills are positively related to their parents’ socioeconomic status^38^. The wealth index is positively associated with child learning skills in Punjab, Pakistan. The academic rate of success in STEM (science, technology, engineering, and mathematics) subjects is less for children of economically deprived backgrounds^39^. The chances of success for children who belonged to the highest quartile are 8 times higher as compared to those who belonged to the lowest quartile^18^.

Regional disparity is obvious for both reading and numeracy skills in this research. Regional differences in learning are suppressed when a model is fitted using only socio-economic and demographic factors. When controlled for both child background and parental involvement factors, differences become more obvious. Fundamental reading abilities are higher in all divisions as compared to Lahore. It is an amazing finding as the Lahore division is called the educational hub of Punjab. Children who belonged to Bahawalpur had the highest chances of adequate reading skills. Children who belonged to Gujranwala and Rawalpindi had shown poor performance in numeracy skills as compared to Lahore.

Parental involvement and the learning environment at home have a positive influence on the learning skills of children. Parental support enhances children’s educational attainment. Children’s engagement in various learning activities like reading books at home increases their understanding of language and polishes their reading skills^40^. Parental engagement in the learning process had a proven positive impact on the educational achievements of children in developed countries^41^. This engagement can be, to remain in contact with the teachers and school administration or participate in school activities^42^. This impact may vary due to cultural norms and the economic status of developing countries.

Parents who attended school to discuss the progress of children discussed educational issues and received report cards of children had a positive impact on their children’s numeracy skills. Although the effect is strong on the performance of children in numeracy skills, only for children whose parents had attended school to discuss child progress. Participation of parents in school celebrations and in financial meetings of school has no positive effect on the performance of their children in basic numeracy skills. A mixed effect of parental involvement is also observed in the literature^43^. Moreover, this research revealed a direct association between reading skills and parental involvement (in assisting school homework, meetings to discuss financial matters, attending school celebrations and discuss progress of children).

## Conclusion

Reading skills of children are observed better for children who are blessed with no functional disabilities. Maternal education has also played a key role in the reading performance of children. Mothers with a higher level of education have a significant positive contribution toward children reading abilities and foundational numeracy skills. Children who had no access to books for reading in appropriate language had a negative impact on their reading skills. The wealth index had an important role on children reading performance but the strength of the effect diminished when adjusted for parental involvement factors. Rich children had predominantly higher possibilities of good numeracy skills than poor children. Parent involvement had a mixed effect in the current study. But results obtained from the behavior of these factors can be deduced generally that parent’s involvement improves children’s academic performance. Children who belonged to Gujranwala and Rawalpindi divisions had ill performed in numeracy skill assessment tests when rest of factors were controlled. Best performance in the reading assessment was found for children residing in Bahawalpur, followed by Sahiwal, Faisalabad, Multan and Rawalpindi.

### Policy implication

Following policy implications are suggested on the basis of the findings of the study.

The Government is recommended to allocate resources for the capacity building of girls’ school in Punjab on a priority basis as the present generation of girls are the future mothers who can have a significant positive impact on their children learning skills. Schools with the help of the government and the community should establish libraries equipped with the books on variety of subjects to cater the reading taste of children. It will help in improving the reading abilities of children.

Punjab Government should continuously monitor the performance and teaching quality of mathematics teachers in Gujranwala and Rawalpindi divisions. Surveys should be conducted in both districts to investigate the reasons for ill performance of children in the numeracy skills. Bahawalpur division may be taken as a case study or model for good performance of children in reading skills and good practices can be shared with other schools of different districts.

Regular parent teacher should be ensured in every school.

## Limitations

The Study is at provisional level not national level. MICS collects data for different provinces of Pakistan. The Study is carried out only for Punjab using most recent wave i.e. MICS 2017–2018 Punjab as for all provinces data for the recent wave has not been released yet. Only those factors were accounted that were available in MICS 2017–2018. Two parental involvement factors i.e. PR7 (School has a governing body in which parents can participate) and PR8 (Attended PTA/SMC meeting in the last 12 months) are not included in the study due to the high percentage of missing values.
